# Recurrent Evolution of Melanism in South American Felids

**DOI:** 10.1371/journal.pgen.1004892

**Published:** 2015-02-19

**Authors:** Alexsandra Schneider, Corneliu Henegar, Kenneth Day, Devin Absher, Constanza Napolitano, Leandro Silveira, Victor A. David, Stephen J. O’Brien, Marilyn Menotti-Raymond, Gregory S. Barsh, Eduardo Eizirik

**Affiliations:** 1 Laboratório de Biologia Genômica e Molecular, Faculdade de Biociências, Pontifícia Universidade Católica do Rio Grande do Sul (PUCRS), Porto Alegre, Brazil; 2 HudsonAlpha Institute for Biotechnology, Huntsville, Alabama, United States of America; 3 Laboratorio de Ecología Molecular & Instituto de Ecologia y Biodiversidad, Departamento de Ciencias Ecológicas, Facultad de Ciencias, Universidad de Chile, Santiago, Chile; 4 Jaguar Conservation Fund, Instituto Onça-Pintada, Mineiros, Goiás, Brazil; 5 Basic Research Laboratory, Frederick National Laboratory, Center for Cancer Research, National Cancer Institute, Frederick, Maryland, United States of America; 6 Theodosius Dobzhansky Center for Genome Informatics, St. Petersburg State University, St. Petersburg, Russia; 7 Instituto Pró-Carnívoros, Atibaia, São Paulo, Brazil; Texas A&M University, United States of America

## Abstract

Morphological variation in natural populations is a genomic test bed for studying the interface between molecular evolution and population genetics, but some of the most interesting questions involve non-model organisms that lack well annotated reference genomes. Many felid species exhibit polymorphism for melanism but the relative roles played by genetic drift, natural selection, and interspecies hybridization remain uncertain. We identify mutations of *Agouti signaling protein (ASIP)* or the *Melanocortin 1 receptor (MC1R)* as independent causes of melanism in three closely related South American species: the pampas cat (*Leopardus colocolo*), the kodkod (*Leopardus guigna*), and Geoffroy’s cat (*Leopardus geoffroyi*). To assess population level variation in the regions surrounding the causative mutations we apply genomic resources from the domestic cat to carry out clone-based capture and targeted resequencing of 299 kb and 251 kb segments that contain *ASIP* and *MC1R*, respectively, from 54 individuals (13–21 per species), achieving enrichment of ~500–2500-fold and ~150x coverage. Our analysis points to unique evolutionary histories for each of the three species, with a strong selective sweep in the pampas cat, a distinctive but short melanism-specific haplotype in the Geoffroy’s cat, and reduced nucleotide diversity for both ancestral and melanism-bearing chromosomes in the kodkod. These results reveal an important role for natural selection in a trait of longstanding interest to ecologists, geneticists, and the lay community, and provide a platform for comparative studies of morphological variation in other natural populations.

## Author Summary

Color polymorphism in closely related animal species provides an opportunity to study how the balance between natural selection and genetic drift shapes the evolution of appearance and form. The cat family, Felidae, is especially interesting; 13 of 37 extant species exhibit polymorphism for melanism, but evidence for any adaptive role is lacking, in part because the potential benefits of melanism to felid predators are not clear, and in part because the tools for genomic analysis of natural populations are limited. We identify the mutations responsible for melanism in three closely related South American wild felids, the pampas cat, the kodkod, and Geoffroy’s cat, then adapt a new approach for targeted genome sequencing to characterize molecular variation in the region surrounding each melanism mutation. We find that each mutation has developed independently, with strong evidence for natural selection in the black pampas cat, and reduced genetic variation in the entire population of kodkods. Our results demonstrate that some “black cats” are black not by chance, but by selection for a mutation that provides increased fitness.

## Introduction

Color variation in natural populations is a useful entry point to investigate the evolution of mammalian phenotypic diversity. Pigmentary differences can be readily observed and quantified; much is known about the underlying biochemistry and cell biology, and there are many examples of apparent convergent evolution. Fundamental questions such as the relative roles played by regulatory vs. protein-coding variation, the phylogenetic origin of similar phenotypes shared among different lineages, and the potential impact of pleiotropic mutations can all be explored from a molecular genetic perspective [1–3].

Mammalian color diversity is particularly apparent in the Felidae, with a wide range of patterns and base colors represented among 37 species with a common ancestor ~11 million years ago [4,5]. In addition to color patterns such as rosettes, stripes, or spots, two characteristic pigmentary phenotypes appear in multiple species [6,7]. The ticked phenotype, a brushed appearance that hides dark tabby markings, is characteristic of 4 wild species (the lion, puma, caracal, and jaguarundi), and melanism, a black coat with residual or “ghost” dark tabby markings in some individuals, has been described in 13 wild species (see [8] for references). Felid melanism is especially interesting because it is polymorphic within each species, but its possible adaptive significance has received little attention to date [9]. In rodents and in birds, an adaptive role of melanism is well-established from ecological and field studies [1,2,10,11], but the evolutionary forces that underlie melanism in larger mammals are less clear, with examples of introgression in the case of North American black wolves [12], and a founder effect in the case of Malaysian black leopards [13]. A recent study by Allen et al. [9] found that the presence of melanism across felid species is correlated with habitat and/or behavioral diversity, and suggested a common underlying mechanism of disruptive selection.

A particularly interesting group of animals from this perspective is an endemic lineage of Neotropical wild cats that includes multiple species exhibiting melanism as a naturally occurring phenotypic variant. This lineage comprises eight species of small cats belonging to the genus *Leopardus*, which diverged from each other after the colonization of South America by a common ancestor ~3 million years ago (MYA) [4,5]. In three of these species, the pampas cat (*L. colocolo*), the kodkod (*L. guigna*, referred to locally as the güiña or hüiña in Chile or Argentina, respectively), and Geoffroy’s cat (*L. geoffroyi*), melanistic individuals comprise 20% or more of the population in some areas [14–17]. Inter-species hybridization within *Leopardus* has been reported [18,19]; thus, high frequencies of melanism within these species could reflect introgression, an ancient trans-specific polymorphism, or independent evolution after divergence from a common ancestor.

To distinguish among these possibilities and to better understand how similar phenotypes evolve in closely related felids, we used massively parallel targeted resequencing to delineate the molecular cause and population genetic history of melanism mutations in each of the three species. The closest reference genome to *Leopardus* spp. is that of the domestic cat, with a last common ancestor ~6 MYA. As an alternative to oligonucleotide capture hybridization, which is constrained to protein-coding regions of closely related species [20], we adapted an approach in which large insert clones (from bacterial artificial chromosome or fosmid vectors) are used as a template to generate biotinylated RNA hybridization probes for solution hybridization capture of homologous segments in the three species. This approach, termed CATCH-Seq (for “Clone Adapted Template Capture Hybridization”) was developed to assess dense human genetic variation in regions that are otherwise difficult to assess by other methods of genotyping [21], e.g. the MHC, but, as shown here, can be extended to facilitate resequencing of targeted genomic regions in any organism for which clone-based reagents from a related species are available.

We identify different molecular causes of melanism in the pampas cat, the kodkod, and the Geoffroy’s cat. Haplotype-based analyses point to distinctive population histories in all three species, with one—the pampas cat—exhibiting strong evidence of selection for melanism. Our results have implications for pigmentary biology and genetics, and yield new insight into the evolution of wild felids.

## Results

### Populations and samples

**Fig 1:**
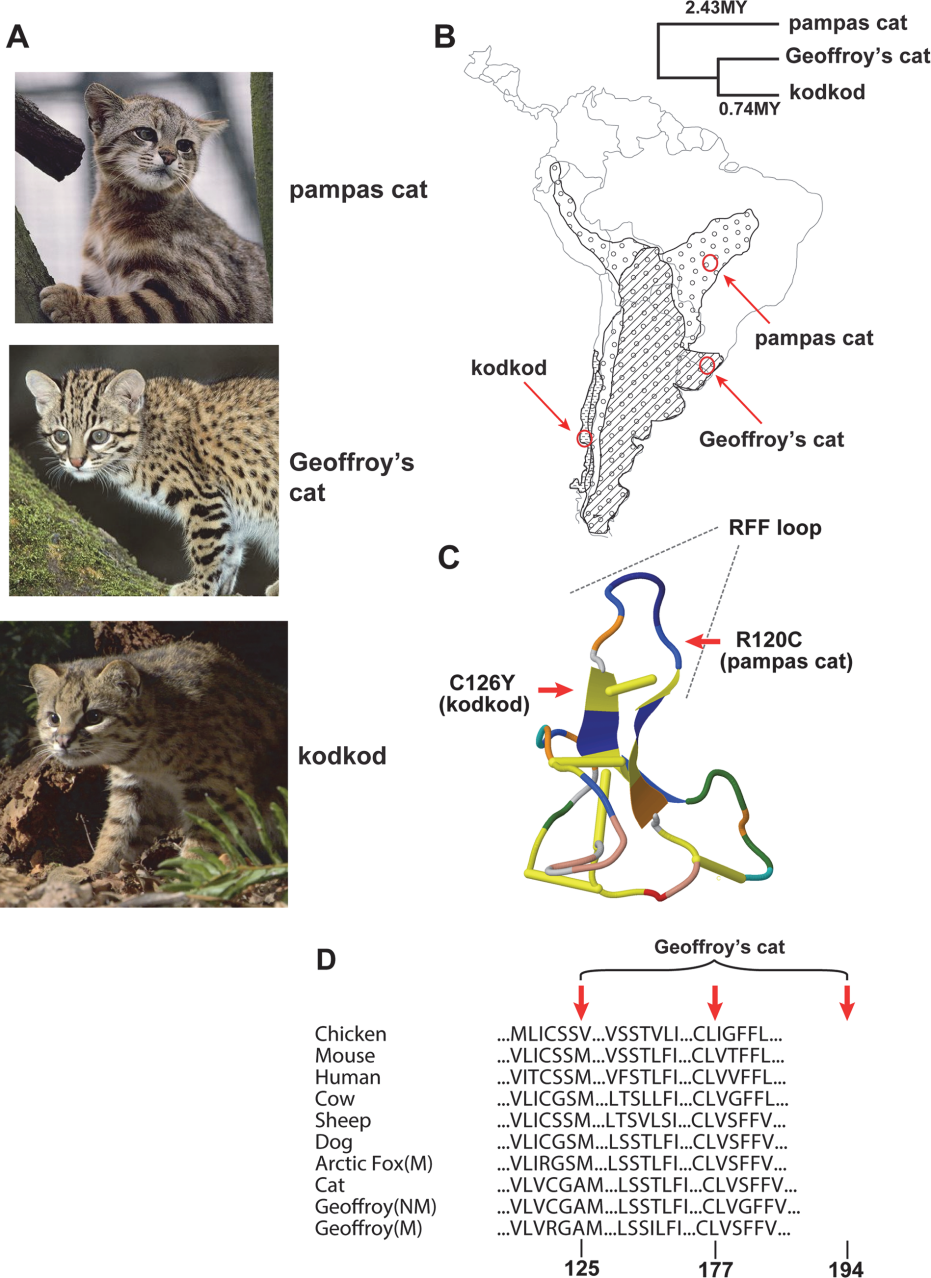
Melanism mutations and phenotypes in three *Leopardus* species (A) Color phenotypes of the pampas cat (*L. colocolo*), Geoffroy’s cat (*L. geoffroyi*), and kodkod (*L. guigna*). Tabby markings in the non-melanistic (NM) forms shown here are obscured in the melanistic (M) forms. (B) Geographic distribution (modified from [7]); red circles represent the approximate origin of samples as described in the text. Right upper panel indicates phylogenetic relationships and estimated divergence times based on [4] (C, D) Location of missense variants in *ASIP* (kodkod, pampas cat) and *MC1R* (Geoffroy’s cat). (C) The kodkod and pampas cat variants are predicted to disrupt the three dimensional structure of a disulfide-stabilized loop in ASIP that is critical for MC1R binding [30]. (D) The Geoffroy’s cat melanism allele carries three missense variants in MC1R; C125R is likely to be causative as described in the text.

The pampas and Geoffroy’s cats are widely distributed through South America, while the kodkod is geographically more restricted (Fig. 1). All three species, which diverged from a common ancestor ~2.5 MYA, are relatively small, inhabit savanna and grassland areas as well as temperate rainforests (particularly the kodkod), and prey on small mammals, birds, and/or amphibians and reptiles. To mitigate against confounding effects of population structure, we sampled individuals from well-defined geographic regions (Fig. 1) in Emas National Park (Brazil), the southern region of Rio Grande do Sul (Brazil), and Chiloé Island (Chile), where the frequencies of melanism are 25%, 20%, and 29% for the pampas cat, Geoffroy’s cat and kodkod, respectively (Table 1, Table S1 in S1 Data).

**Table 1:** Genotype, allele, and phenotype frequencies for melanism variants.

Species	Phenotype	Genotype^a^			Melanism allele frequency	Predicted phenotype frequency^a^	Observed phenotype frequency^b^
		ASIP					
		+/+	+/R120C	R120C/R120C			
Pampas cat	Ancestral	2	6	0	0.71	0.50	0.25
	Melanistic	0	0	9			
		+/+	+/C126Y	C126Y/C126Y			
Kodkod	Ancestral	5	6	0	0.50	0.25	0.29
	Melanistic	0	0	5			
		MC1R					
		+/+	+/C125R	C125R/C125R			
Geoffroy’s cat	Ancestral	16	0	0	0.15	0.28	0.20
	Melanistic	0	7	0			

^a^ We use R120C (in ASIP), C126Y (in ASIP), and C125R (in MC1R) to refer to melanism alleles in the pampas cat, the kodkod, and Geoffroy’s cat, respectively. One pampas cat lacking information on geographic origin (Lco-030, Tables S1, S5 in S1 Data) is excluded from the results shown here. Predicted phenotype frequencies assume Hardy-Weinberg equilibrium.

^b^ For the pampas cat, observed phenotype frequencies are based on camera trapping data obtained by one of us (17 of 68 images, L.S.) from the same population. For the kodkod, observed phenotype frequencies are based on ref. (Sanderson 2002). For Geoffroy’s cat, observed phenotype frequencies are based on a combination of wild-caught and road-killed animals from the same population.

### Melanism and pigment type-switching: mutations in *Agouti signaling protein* (*ASIP*) and *Melanocortin 1 receptor* (*MC1R*)

CATCH-Seq is best suited to explore sequence variation once specific regions of interest are identified; therefore we initiated molecular analyses of melanism by conventional capillary-based sequencing of candidate genes. In mammals, melanism represents a shift in the balance between red-yellow pheomelanin and brown-black eumelanin, controlled by the activity of a melanocyte-specific G protein-coupled receptor, the Melanocortin 1 receptor (MC1R) [22–25]. The most common causes of melanism mutations are gain-of-function alterations in *MC1R*, or loss-of-function alterations in *ASIP*, which encodes Agouti signaling protein, a paracrine signaling molecule that inhibits MC1R signaling [26]. Consistent with the nature of the mutations, melanism caused by *MC1R* mutations is dominantly inherited, while melanism caused by *ASIP* mutations is recessively inherited; however, the inheritance pattern of melanism in pampas, Geoffroy’s cat, and the kodkod is not known. (Additional, less frequent causes of melanism in some mammals include mutations of beta-defensin 103 [an alternative ligand for MC1R] [27], Attractin [an accessory receptor for ASIP] [28], or Mahogunin [an E3 ubiquitin ligase that acts upstream of the MC1R] [29]).

Using the domestic cat genome as a starting point for the design of oligonucleotide PCR primers, we determined the protein-coding sequence of *ASIP* and *MC1R* in 18 pampas cats (10 melanistic), 23 Geoffroy’s cats (7 melanistic), and 16 kodkods (5 melanistic). Animals were selected from specific geographic areas as described above, and collection was independent of coat color phenotype (Table S1 in S1 Data). In each species, we identified missense alterations whose molecular nature, pattern of association, and apparent mode of inheritance made a compelling case for them being the cause of melanism (Table 1, Table S4 in S1 Data, Fig. 1).

In both the pampas cat and the kodkod, mutations in *ASIP* were identified that are predicted to cause a loss-of-function, and homozygosity for these mutations was completely associated with melanism. In the pampas cat, an inferred Arg to Cys substitution in the C-terminal region of ASIP (p.R120C) lies in the critical RFF loop (e.g. a triplet motif made of one arginine and two phenylalanine residues), required for binding to the MC1R [30], and also creates an odd number of cysteine residues, predicted to interfere with folding of the disulfide-rich domain. Similarly, in the kodkod, an inferred Cys to Tyr substitution in ASIP (p.C126Y) affects the key disulfide bond that stabilizes the RFF loop [30]. All melanistic pampas cat individuals were homozygous for *ASIP* ^*R120C*^ (Chi-square = 18, p<0.005) and all melanistic kodkod indivduals were homozygous for *ASIP* ^*C126Y*^ (Chi-square = 16, p<0.005). No other *ASIP* coding sequence variants were identified in the pampas cat or in the kodkod (Table S5 in S1 Data); four intraspecific variants in *MC1R* were present in the pampas cat, but none of them exhibited an association with melanism (Table S4 in S1 Data).

In Geoffroy’s cat, we identified an *MC1R* mutation as the likely cause of melanism. All melanistic individuals were heterozygous for four variants that predicted three nonsynonymous substitutions, p.C125R, p.T177I, and p.G194S (Table S6 in S1 Data). All individuals were either homozygous for the ancestral alleles (125Cys, 177Thr, 194Gly) or heterozygous for the derivative alleles (125Arg, 177Ile, 194Ser); we observed complete association of heterozygosity for the derivative alleles with melanism (Chi-square = 16, p<0.005). In the laboratory mouse, a Cys to Arg substitution at the site homologous to felid residue 125 causes constitutive activation of the receptor [31], and the exact same change is thought to be responsible for melanism in the Alaska silver fox [32]; therefore, we consider C125R to be the likely causative alteration in the Geoffroy’s cat.

Transmission of melanism has not been described in these species, but the amino acid substitutions and patterns of association predict that melanism in the pampas cat and kodkod is recessively inherited, and that melanism in the Geoffroy’s cat is dominantly inherited. Frequencies of the melanism alleles are 0.71, 0.5, and 0.15 for the pampas cat, the kodkod, and Geoffroy’s cat, respectively, and are consistent with the field-based assessments of melanism frequency in the populations from which each sample was drawn (Table 1). Like other wild felids that occur at low densities, small sample sizes constrain the power of studies based on estimates of genotype frequency. With that caveat, a relatively high prevalence of melanism due to independent mutations in these three species suggests that the phenotype is not deleterious [33].

### CATCH-Seq libraries: construction, analysis, and validation

**Fig 2:**
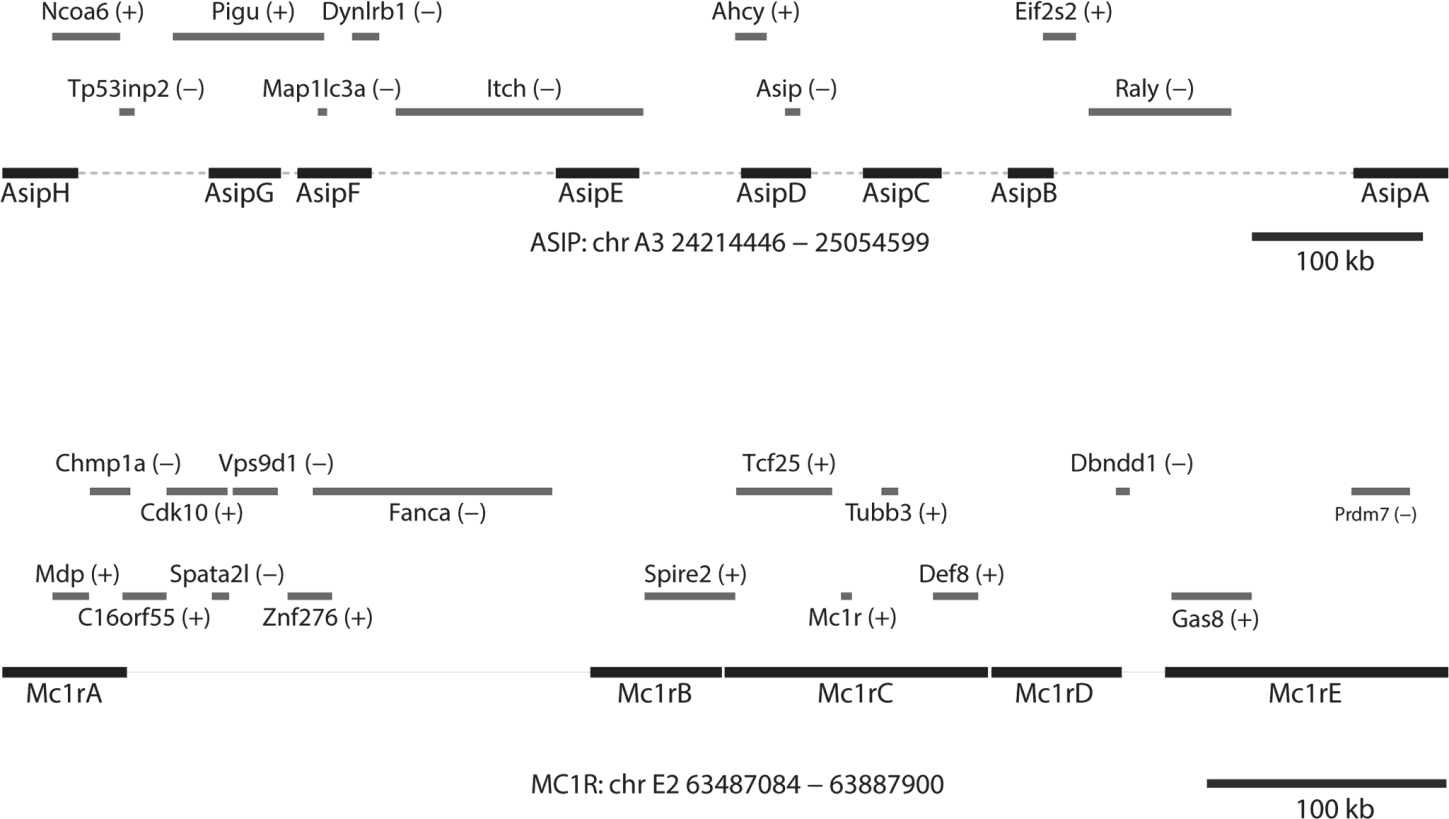
Positions of fosmids and neighboring genes at melanism loci. Contig lengths and spacing are drawn to scale (see Table S7 in S1 Data for individual coordinates); sense or antisense transcription, relative to the chromosomal orientation, is indicated with (+)/(-).

Originally developed as a cost-effective way for targeted resequencing of human regions that are highly polymorphic, clone-based target capture is also uniquely suited for studying non-model organisms for which large insert genomic libraries from a closely related organism are available. To survey genetic variation within and around *ASIP* and *MC1R* in *Leopardus* spp., we identified a series of fosmids from the domestic cat reference genome that contain and surround each locus (Fig. 2). For *ASIP*, we used 8 fosmids that contain 299 kb of DNA within an 842 kb interval; for *MC1R*, we used 5 fosmids that contain 251 kb of DNA within a 409 kb interval (Fig. 2, Table S7 in S1 Data). Illumina paired-end libraries were constructed from sheared genomic DNA of 57 animals (Table S1 in S1 Data). From 54 libraries that passed quality control, material selected by CATCH-Seq using biotinylated probes prepared from the 13 fosmids was sequenced in a multiplexed format; a total of 1,053,331,306 paired-end reads (50 bp) were collected.

Summary alignment and mapping statistics are presented in Table 2. The current domestic cat assembly (Felis_catus-6.2) spans 2.43 Gb and represents ~14x Q20 coverage, but has not yet been fully annotated. Approximately 70% of our sequence reads aligned to the cat genome; of these, ~58% aligned to one of the *ASIP* or *MC1R* target fosmids considering the entire sequence of the fosmids, and ~7% aligned to the *ASIP* or *MC1R* target considering the repeat-masked sequence of the fosmids, consistent with an enrichment value of ~500–~2500-fold (Table 2, Table S9 in S1 Data).

**Table 2:** Alignment coverage summary for CATCH-Seq libraries.

	Size (kb)	No. of aligned reads^a^	Theoretical coverage^a^	Enrichment
Cat Reference	2430000	699,823,384	0.79 (A)	
Target (total)^b^	550.61	406,653,630	2051 (B)	(B/A) 2564
Target (masked)^b^	329.82	46,707,320	393 (C)	(C/A) 492

^a^ Datasets include 150 bp PE and 100 bp PE Illumina reads; theoretical coverage per sample was calculated assuming an average of 75 bp aligned sequence per end, e.g. (699,823,384 x 150)/(54 x 2.43 x 10^9^). The number of aligned reads shown here represents the results from all 54 *Leopardus* spp. libraries; results for each species are given in Table S9 in S1 Data.

^b^ Target sizes represent the sum of the 13 fosmid sizes before and after use of RepeatMasker with default parameters (Table S8 in S1 Data).

The mean coverage of each fosmid was 150x, with 71%–99% (mean, 92%) of non-repetitive sequence covered at > 10x (Table S10 in S1 Data). Nucleotide divergence between the domestic cat and *Leopardus* spp. was ~1–2%, making the domestic cat unsuitable as a reference for variant-calling; therefore, we used SAMtools to develop a consensus sequence for each species separately (combining melanistic and non-melanistic individuals within each species). The species-specific consensus sequence was then used to establish high-confidence variant calls for each individual according to best practices for the Genome Analysis Toolkit, and likely haplotype structure was inferred using Beagle.

We identified 149, 383, and 474 SNPs in the kodkod, pampas cat, and Geoffroy’s cat, respectively, which yield estimates of percent nucleotide diversities of 0.0180 (kodkod), 0.0451 (pampas cat), and 0.0572 (Geoffroy’s cat) (Table S11 in S1 Data). The different nucleotide diversities are not artifacts of ascertainment, since alignment statistics and coverage are similar for the three species (Supplemental Tables S9, S10). (Coverage of fosmids in the Geoffroy’s cat was not as complete, ~15x, as in the kodkod (~35x) or pampas cat (~20x) [Table S10 in S1 Data], but more SNPs were identified in Geoffroy’s cat than in the other two species). Instead, the differences in nucleotide diversity likely reflect species-specific demographic histories as described below.

**Fig 3:**
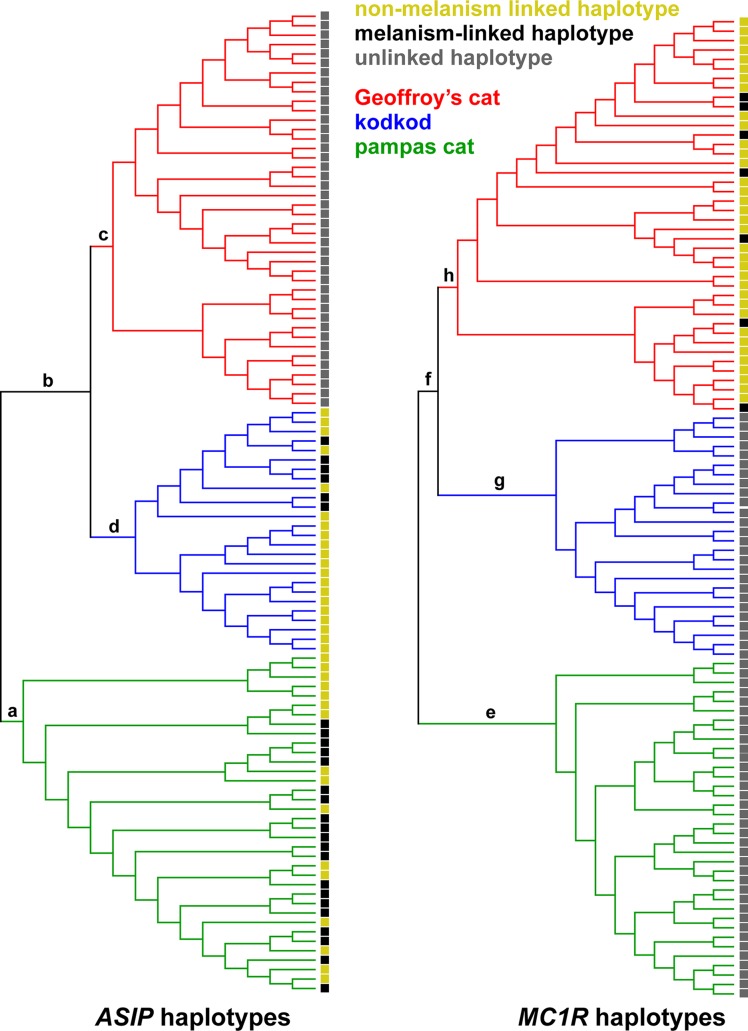
Phylogenetic relationships among chromosomes for each locus inferred with BEAST2 [34]. Posterior probabilistic support for the main branches of the maximal clade credibility trees are: a – 0.987, b – 0.991, c – 0.988, d – 0.988, e – 0.999, f – 0.999, g – 0.995, h – 0.992. Colored squares on the right of each tree indicate whether each haplotype is linked to (*e.g.* contains) a melanism-associated mutation.

To help validate our pipeline, and to further explore the pattern of variant distribution between and within species, we carried out a phylogenetic analysis for the *ASIP* and *MC1R* regions using a Bayesian Markov Chain Monte Carlo (MCMC) approach [34], in which individual chromosomes for each locus were clustered and the clade credibility for the major nodes was examined. Although this approach does not account for recombination and therefore is not informative with regard to gene genealogy within species, the tree topologies should approximate evolutionary relationships among species, and also provide some assessment of quality control with regard to potential population structure as well as the accuracy of species identification. As depicted in Fig. 3, the posterior clade probability for the nodes representing the divergence of the three species is very high for both *ASIP* and *MC1R* loci, with basal relationships that recapitulate the known topology in which the kodkod and Geoffroy’s cat are more closely related to each other than to the pampas cat. For the pampas cat and Geoffroy’s cat, chromosomes bearing melanism mutations (*ASIP* for pampas cat, *MC1R* for Geoffroy’s cat) do not cluster together; for the kodkod, there is some clustering of melanistic *ASIP* chromosomes that may reflect recent population history, as described below.

### Molecular evolution of melanism mutations

A fine-scale view of haplotype patterns and nucleotide diversity allows additional insight into the evolutionary history underlying felid melanism. Positive selection is expected to give rise to reduced nucleotide diversity at the melanism locus (*ASIP* in the pampas cat and kodkod, *MC1R* in Geoffroy’s cat), but not at the “control” locus (*MC1R* in the pampas cat and kodkod, *ASIP* in Geoffroy’s cat).

**Fig 4:**
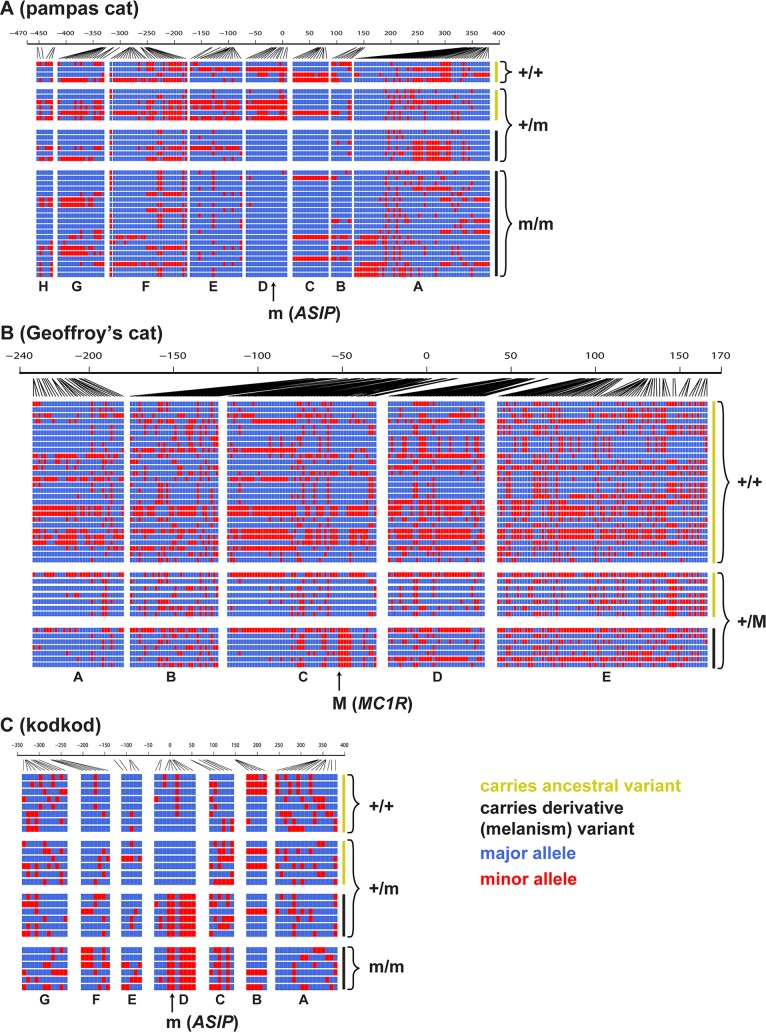
Haplotype structures for melanism loci for pampas cat (A), Geoffroy’s cat (B), and kodkod (C), arranged according to whether they carry an ancestral (yellow) or derivative (black) allele at the causative position, and whether they were observed in a non-melanistic (+/+, +/m) or melanistic (m/m, +/M) individual. Major and minor alleles are depicted in blue and red respectively, and genomic coordinates are represented in kb upstream or downstream from the causative position. Letters indicating the fosmids that correspond to each targeted segments are shown at the bottom of each plot.

Fig. 4 depicts the pattern of variation for individual chromosomes at the *ASIP* (pampas cat and kodkod) and *MC1R* (Geoffroy’s cat) loci, grouped according to whether the chromosome carries an ancestral or derivative allele, and whether it was observed in a melanistic (m/m or +/M) or a non-melanistic (+/+ or +/m) animal. At the level of individual haplotypes, multiple variants in the pampas cat define a large haplotype block (Fig. 4A) that extends throughout the AsipD fosmid and that contains the *ASIP* melanism mutation. Haplotype diversity is also reduced for melanism-bearing chromosomes in the pampas cat: within the AsipD fosmid, there are 3 haplotypes among 26 melanism-bearing chromosomes compared to 8 haplotypes among 10 ancestral chromosomes. In the Geoffroy’s cat, four variants within the Mc1rC fosmid delineate a ~1kb haplotype block almost perfectly associated with melanism (Fig. 4B); beyond this central core, there is no obvious distinction in haplotype structure or haplotype diversity between chromosomes that carry melanism mutations and those that do not. Haplotype patterns in the kodkod reveal striking differences between melanism-bearing and ancestral chromosomes, but relatively little within-group difference; in the AsipD fosmid, there are 3 haplotypes among 10 melanism-bearing chromosomes, and 5 haplotypes among 14 ancestral chromosomes (Fig. 4C).

**Fig 5:**
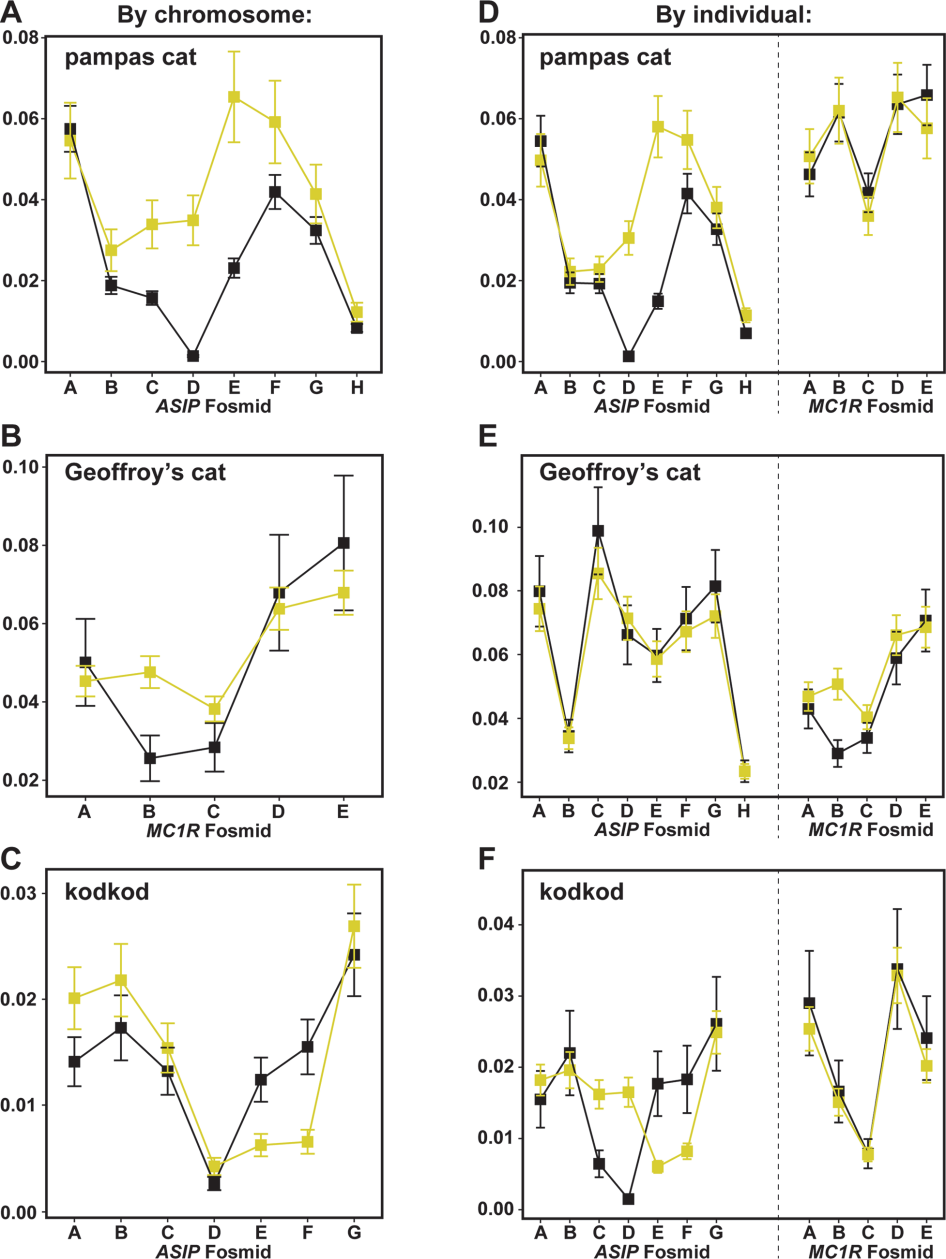
Nucleotide diversity calculated for ancestral (yellow) vs. melanism-bearing (black) chromosomes, and for individuals according to their phenotype (melanistic, black vs. non-melanistic, yellow), for pampas cat (A, D), Geoffory’s cat (B, E) and kodkod (C, F). As described in the text, the latter approach allows comparison between loci within each species, illustrating, e.g. that patterns of nucleotide diversity are similar between melanistic individuals for the “control” locus (*ASIP* in the Geoffroy’s cat, *MC1R* in the pampas cat and kodkod). Error bars indicate standard error of the mean (SEM).

We next compared nucleotide diversity between melanism-bearing and ancestral chromosomes (Fig. 5A, B, C), using fosmid origin (Fig. 2) as a window for proximity to the causative variant. In the pampas cat, melanism-bearing chromosomes exhibit a clear signature of selection (Fig. 5A), with reduction of nucleotide diversity that extends across a ~500 kb interval surrounding the causative variant (*ASIP* fosmids AsipB, AsipC, AsipD, AsipE, AsipF, AsipG), but levels of nucleotide diversity similar to ancestral chromosomes in flanking fosmids (*ASIP* fosmids AsipA, AsipH). In the Geoffroy’s cat (Fig. 5B), melanism-bearing chromosomes also exhibit a window of reduced nucleotide diversity in a region ~50 kb upstream of the causative variant (*MC1R* fosmid Mc1rB), although the extent of the difference is not as striking as in the pampas cat. In the kodkod, nucleotide diversity is similarly low for both melanism-bearing and ancestral chromosomes in the AsipD fosmid; in two adjacent fosmids (AsipE, AsipF), levels are modestly higher for melanism-bearing compared to ancestral chromosomes (Fig. 5C).

We also calculated nucleotide diversity for melanistic compared to non-melanistic individuals (Fig. 5D, E, F); although less sensitive for evaluating differences in derivative *vs.* ancestral chromosomes (since non-melanistic pampas cats and kodkods are a mixture of +/m and +/+), this approach allows a direct comparison of nucleotide diversity at a locus unlinked to the causative variant (*MC1R* for the pampas cat and kodod; *ASIP* for the Geoffroy’s cat). There are no systematic differences between melanistic and non-melanistic individuals for loci that are unlinked to the causative variant, whereas differences observed for the pampas cat and Geoffroy’s cat in a by-chromosome analysis of the causative locus (Fig. 5A, B) persist in a by-individual analysis of the causative locus (Fig. 5D, E).

**Fig 6:**
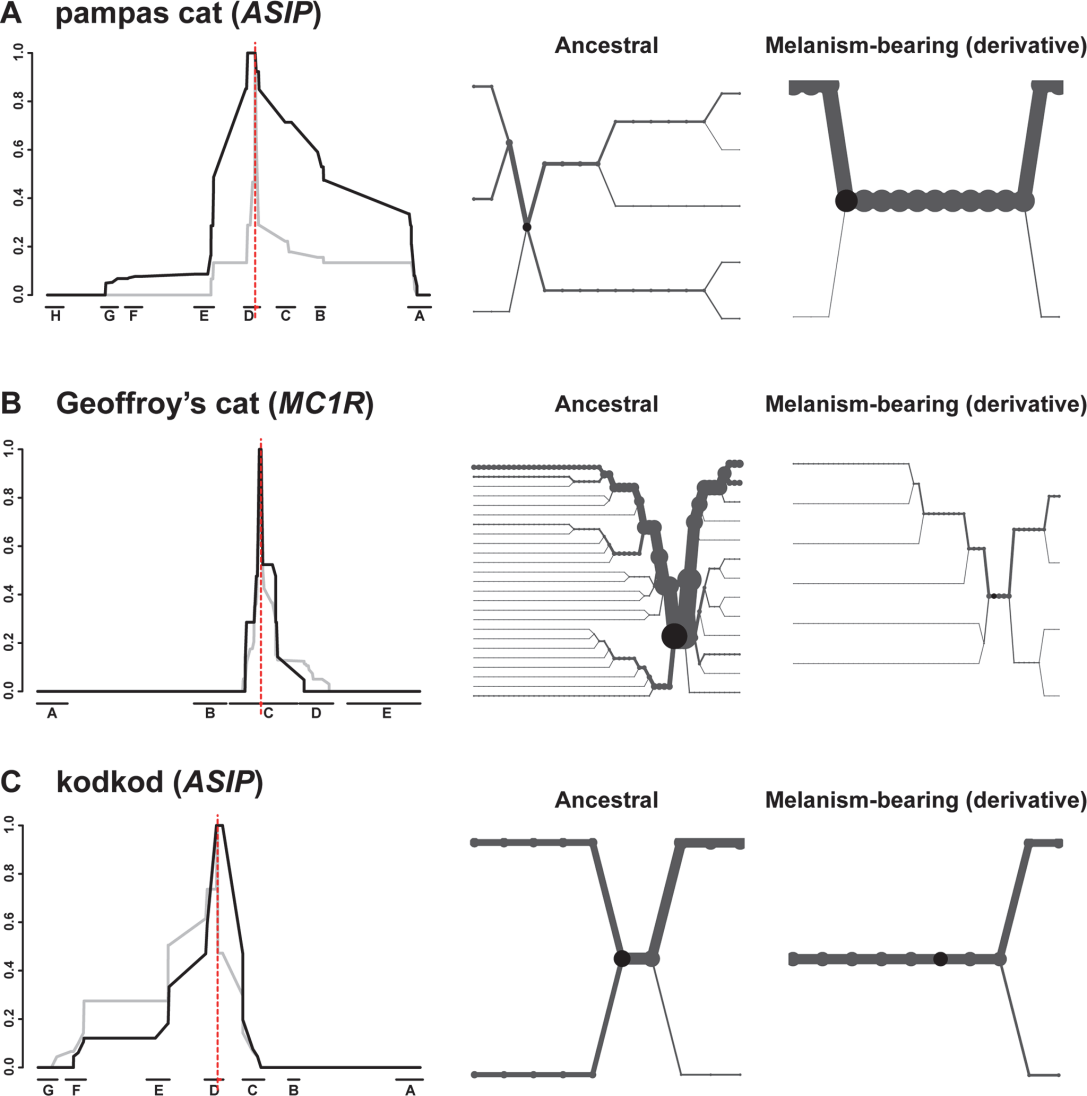
Long-range haplotype analysis for pampas cat (A), Geoffory’s cat (B) and kodkod (C) respectively. The left-hand side depicts relative EHH according to distance from the causative variant (vertical red line) plotted on the y-axis for ancestral (grey) vs. melanism-bearing (black) chromosomes, as a function of genomic location plotted on the x axis, indicated according to fosmid identity (Fig. 2). The right-hand side depicts haplotype bifurcation plots with origins at the causative variant: branches represent haplotype divergence, and the thickness of the lines is proportional to the number of chromosomes.

A signature of selection in the pampas cat is also readily apparent from graphs of extended haplotype homozygosity (EHH) and haplotype bifurcation diagrams (Fig. 6A); EHH for melanism-bearing chromosomes in the pampas cat extends for hundreds of kilobases with relatively few bifurcations. By contrast, the Geoffroy’s cat and the kodkod exhibit similar EHH for ancestral and derivative chromosomes (Fig. 6B, C).

Haplotype diversity, nucleotide diversity, and EHH in the kodkod are unusual: nucleotide diversity graphs for melanism-bearing and ancestral chromosomes intersect and cross within the AsipD fosmid (Fig. 5C), and the EHH graphs in the kodkod are similar for ancestral and melanism-bearing chromosomes (Fig. 5C). Although this pattern does not lend itself to a simple interpretation, genetic drift is likely to be a contributing factor given the overall reduction of nucleotide diversity; 0.018% in the kodkod compared to 0.045% and 0.057% in the pampas and Geoffroy’s cats, respectively.

## Discussion

The application of short-read DNA sequencing technology to longstanding questions in mammalian evolution offers significant opportunities as well as challenges. Morphological variation within a species, or between closely related species, is often hypothesized to serve an adaptive role, a question that can be informed by knowing the molecular basis and evolutionary history of the underlying events. This effort can be challenging, however, in settings where a reference genome does not yet exist. As shown here, our application of a clone-based targeted capture-resequencing strategy reveals distinct evolutionary histories for melanism in three closely related *Leopardus* species. The results have implications for understanding the biological and genetic basis of melanism in other mammals, inform ongoing efforts in conservation biology, and provide a molecular example that can be applied more widely in population and evolutionary genomics.

Melanism is one of the most common color morphs in domestic and natural populations of birds and mammals, and in most species it is caused by a loss-of-function mutation in *ASIP* or a gain-of-function mutation in *MC1R* [3,10,26]. The same now appears to be true in felids; including the three *Leopardus* species presented here, *ASIP* or *MC1R* mutations have now been found to cause melanism in 8 felid species [8,35], a trend that seems likely to continue for the remaining 5 felid species (the jungle cat, the marbled cat, the bobcat, the tigrina, and the serval), in which melanism is recognized but not characterized from a molecular genetic perspective. Loss-of-function mutations in two additional and related pigment type-switching components, *Attractin* and *Mahogunin*, cause melanism in the laboratory mouse or rat, but these mutations also cause brain abnormalities and would probably be subject to negative selection in natural populations [36]. In North American wolves, an unusual gain-of-function alteration in beta-defensin 103 causes dominantly inherited melanism and was acquired by hybridization with domestic dogs; however, beta-defensin variants that affect pigmentation have not been recognized outside of dogs, wolves, or coyotes [12,27].

Interspecies hybridization is not uncommon among *Leopardus* species [19], but we observed no examples of variants that were shared between species. Our results show that at least three independent melanism mutations have occurred during recent evolution of this genus, and have remained polymorphic within each species. Molecular genetic evidence for an adaptive role is strongest for the pampas cat, in which the causative *ASIP* variant lies on a single major haplotype that extends for hundreds of kilobases as part of a selective sweep. However, absence of long haplotype patterns that clearly distinguish ancestral and melanism-bearing chromosomes in the Geoffroy’s cat and kodkod does not exclude the possibility that melanism is adaptive in these species. The Geoffroy’s cat in particular exhibits patterns of variation that deviate from neutral expectation, with all melanism-bearing chromosomes carrying three non-synonymous mutations in *MC1R*-coding sequence, and reduced levels of nucleotide diversity in a region ~50 kb upstream of *MC1R*. Potential selection for melanism in the Geoffroy’s cat may have occurred earlier than in the pampas cat; alternatively, or in addition, a potentially higher recombination rate at *MC1R* than at *ASIP* may have diminished the size of an extended haplotype in the Geoffroy’s cat. Studies of additional Geoffroy’s cat populations may help reveal whether the unusual haplotype pattern associated with melanism reflects a specialized population history in Southern Brazil or a more general aspect of the derivative chromosome.

In the kodkod, haplotype structure is similar between melanism-bearing and ancestral chromosomes, but the patterns of nucleotide diversity are unusual. Furthermore, within the fosmid that carries the causative variant (AsipD), multiple SNPs sort the melanism-bearing and ancestral chromosomes into two different lineages. A recent population bottleneck associated with colonization of Chiloé Island could account for the latter observation, but does not easily explain why ancestral chromosomes exhibit a local reduction of nucleotide diversity anchored by the *ASIP* gene. One intriguing possibility is frequency-dependent balancing selection, such that both melanistic and non-melanistic phenotypes have adaptive value, but only at low frequencies. Larger sample sizes and evaluation of multiple loci would provide additional insight on this hypothesis.

The kodkod is also striking for its very low nucleotide diversity (0.018%), among the lowest described among all mammals [37], which is consistent with assessments from ecological studies indicating that the restricted geographic range of this species coupled with deforestation of Central-southern Chile has reduced its effective population size [14–17,38]. Although it can be challenging to distinguish if loss of genetic diversity is a cause or consequence in any natural population, it is directly related to population reduction.

Melanism is a quintessential example of natural selection in many animals; mechanisms that underlie adaptation can be difficult to discern in larger mammals, although recent work from Allen et al. [9] provides evidence for disruptive selection. In felids, crypsis, presumably as an aid to predation, is thought to drive variation in color patterns, but variation in base color is often postulated to facilitate temperature regulation or response to UV radiation. The latter two mechanisms are oft-cited explanations for Gloger’s rule (correlation between prevalence of darker coloration and equatorial proximity), but are unlikely to explain persistent polymorphism of melanism within *Leopardus* spp.

Our results point to an important role for natural selection in the evolution of melanism for at least one of the analyzed species, but reveal very different genomic signals that likely reflect unique demographic histories, influenced by different selective processes in addition to drift and recombination. Application of similar approaches to other species will extend our knowledge of how natural selection has shaped color variation, and offer new avenues to investigate the evolutionary history of phenotypic diversity.

## Materials and Methods

### Animals and genomes

We studied a total of 57 individuals (18 pampas cat, 16 kodkod, and 23 Geoffroy’s cat) whose origin and phenotype are listed in Table S1 in S1 Data. Biological material was either blood (collected from wild-caught individuals in the context of field ecology studies) or tissue (from road-killed animals encountered during routine wildlife surveys); in all cases, sampling was performed following appropriate national regulations for handling animals and biological materials. DNA was prepared by extraction with phenol/chloroform, and assessed for concentration and quality by fluorometry and agarose gel electrophoresis, respectively.

When this work was initiated, we made use of an annotated but incomplete (1.9x) genome assembly of the domestic cat, felCat3 [39]. During the course of the work, two additional assemblies became available, and all coordinates used in the manuscript now refer to felCat5/Felis_catus-6.2, http://genome.wustl.edu/genomes/detail/felis-catus/.

### Candidate gene genotyping

After PCR amplification, protein-coding exons of *ASIP* [8] and *MC1R* (Table S2 in S1 Data) were analyzed by automated capillary sequencing. We first examined 8 individuals of each species (4 of each phenotype), then extended those results to all available samples (Supplemental Tables S1, S4, S5, S6). Sequencing electropherograms were verified and corrected with Sequencher 4.2 (GeneCodes Corporation), and every potential variant was carefully inspected for confirmation. Homologous nucleotide and amino acid sequences of each gene from additional mammalian species were obtained from GenBank (Table S3 in S1 Data) and aligned using ClustalW.

### Targeted resequencing

DNA libraries for Illumina sequencing were generated using standard protocols. Briefly, 700ng to 2.5ug of input DNA (depending on the level of DNA degradation assessed by agarose gel electrophoresis before shearing) was sheared to a size range of 100–500 bp, and custom inline barcodes were added during adapter ligation for paired-end sequencing.

For target selection, we utilized a set of fosmid libraries that had been previously end-sequenced and mapped to a 3x draft assembly, V17e/felCat4 [40]; identity of all fosmids was verified by PCR of each end.

A detailed protocol for probe preparation, hybridization, and capture is described elsewhere (Day et al., manuscript submitted). Briefly, fosmids were pooled according to their inferred individual mass, a total of 1.5ug of input was sheared to 100–500 bp, and DNA fragments were used to prepare biotinylated RNA with a Megascript T7 kit (Ambion). After DNAse treatment and removal of unincorporated nucleotides, hybridizations were carried out in a volume of 26ul that contained 300 ng of probe and 500ng of library DNA (125ng each of 4 inline barcoded libraries). Selected DNA was recovered with magnetic beads and amplified by PCR (20 cycles using standard Illumina primers). Library 4-plex pools were sequenced as 12-plex library sets on a single Illumina HiSeq 2000 lane.

### Bioinformatics

We used *ASIP* and *MC1R* regions from chromosomes A3 and E2, respectively, as reference sequences, extracted from the Felis_catus-6.2 assembly. The genomic reference was masked for transposable elements and low complexity regions with *RepeatMasker* [41]. To minimize genotyping errors related to inter-species differences in allele structure and distribution, we first mapped all raw sequence reads against the domestic cat reference using the *Burrows-Wheeler Aligner* (*BWA*) with default parameters [42], then used *SAMtools* [43] to compute consensus sequences for these preliminary alignments, and to *de novo* assemble targeted regions in each of the three species separately.

Sequence reads were then remapped to these *de novo* species-specific consensus sequences and subsequently used to for variant calling according to *GATK* best practices guidelines [44]. Briefly, we started by applying alignment quality control procedures available in *GATK* to detect sequence intervals with low quality mappings (*i.e.* possibly related to the presence of sequence variants, such as small insertions or deletions, in subsets of the analyzed samples). In all such cases, a thorough local realignment of the reads was performed to minimize the number of mismatching bases. The *GATK Unified Genotyper* (UG) tool was applied on realigned reads to infer the genotype structure simultaneously across all samples for each species. The raw genotype calls were subjected to a filtering procedure by imposing thresholds on a set of quality criteria, including minor allele frequency MAF>10%, Phred-scaled mapping quality MQ>40, UG quality by depth QD>2 and UG HaplotypeScore>13. The filtered calls were further restricted to a small subset of high quality calls, to satisfy an average variant density threshold of ~1 per 1kb of target sequence. Finally, the *BEAGLE* genetic analysis software package [45] was used to check genotype consistency across all samples of each species and infer the haplotype phase of selected variants.

Curated genotype and haplotype data was then used for downstream analyses, including nucleotide diversity and polymorphism statistics with *DnaSP* 5.10 [46], EHH [47], and generation of median-joining haplotype networks (*Network* 4.5.0.0; http://www.fluxus-engineering.com). Haplotype bifurcation plots in Fig. 5 were generated with *Sweep* 1.1 (http://www.broadinstitute.org/mpg/sweep/index.html) and *rehh* 1.0 [48].

### Data access

DNA sequences reported in this manuscript are publicly available from DRYAD (doi:10.5061/dryad.pq482).

## Supporting Information

S1 Data Supplementary tables containing supporting data. (DOCX)
