# Correction: Recurrent Evolution of Melanism in South American Felids

**DOI:** 10.1371/journal.pgen.1005126

**Published:** 2015-04-10

**Authors:** The PLOS Genetics Staff

Due to an error in the production process, the labels for panel D in Fig. 1 are misaligned. The correct figure is provided here.

The Dryad DOI in the Data Availability Statement links out incorrectly. The correct Dryad link is http://dx.doi.org/10.5061/dryad.pq482.

**Fig 1:**
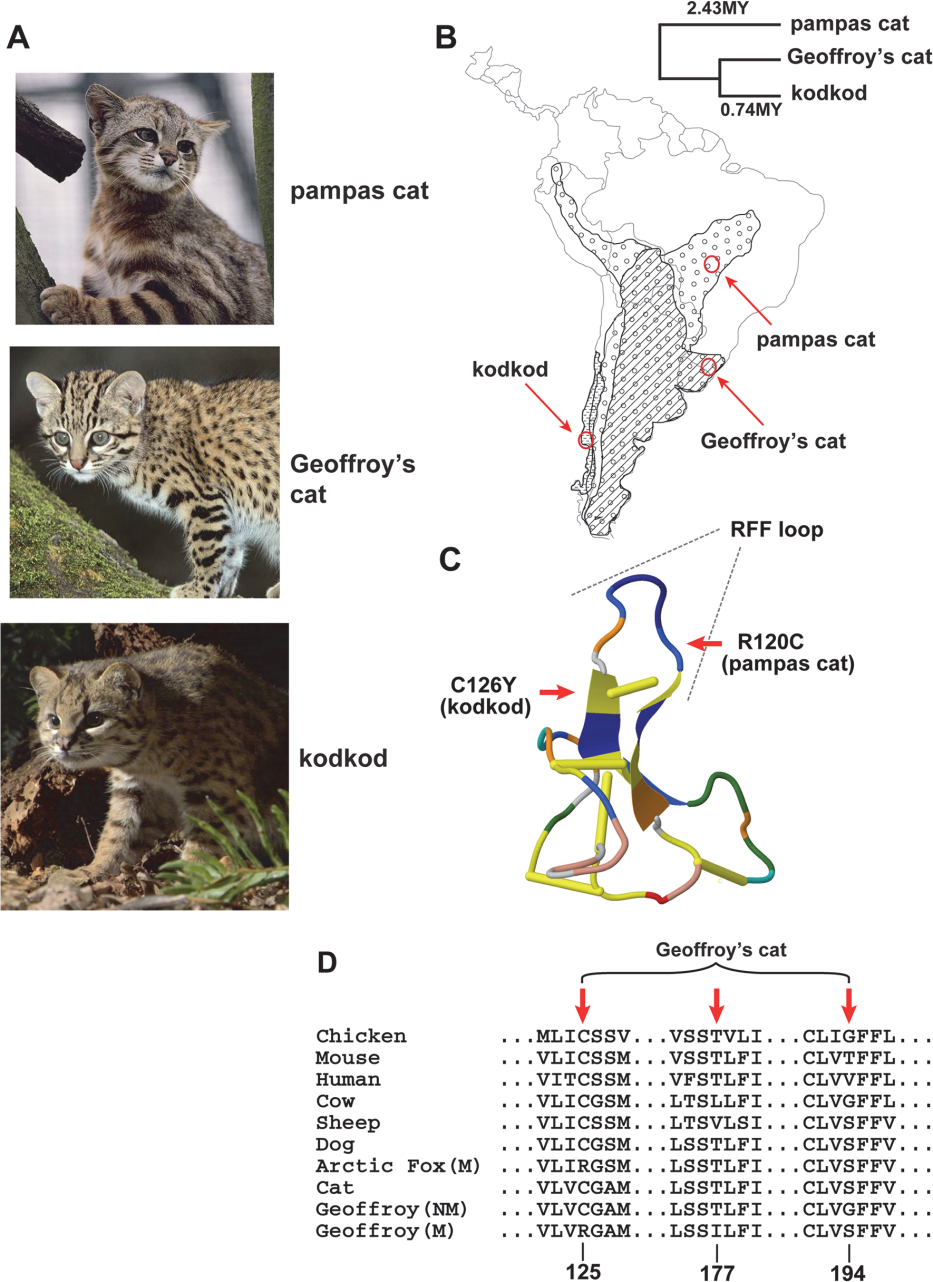
Melanism mutations and phenotypes in three *Leopardus* species. (A) Color phenotypes of the pampas cat (*L*. *colocolo*), Geoffroy’s cat (*L*. *geoffroyi*), and kodkod (*L*. *guigna*). Tabby markings in the non-melanistic (NM) forms shown here are obscured in the melanistic (M) forms. (B) Geographic distribution (modified from [7]); red circles represent the approximate origin of samples as described in the text. Right upper panel indicates phylogenetic relationships and estimated divergence times based on [4] (C, D) Location of missense variants in *ASIP* (kodkod, pampas cat) and *MC1R* (Geoffroy’s cat). (C) The kodkod and pampas cat variants are predicted to disrupt the three dimensional structure of a disulfide-stabilized loop in ASIP that is critical for MC1R binding [30]. (D) The Geoffroy’s cat melanism allele carries three missense variants in MC1R; C125R is likely to be causative as described in the text.
