# Supplementary material for: Recurrent Evolution of Melanism in South American Felids
Source: PLoS Genet. 2015 Feb 19;11(2):e1004892. doi: 10.1371/journal.pgen.1004892 (PMC4335015; doi:10.1371/journal.pgen.1004892)
Supplement: S1 Data — (DOCX) [file pgen.1004892.s001.docx]

**Table S1. List of the analyzed samples and their genotypes at the melanism-related *ASIP* and *MC1R* loci.**

| **Sample ID** | **Geographic Origin** | | **Institution/Contact** | **Phenotype** | **Genotype** | | |
| --- | --- | --- | --- | --- | --- | --- | --- |
|  |  |  |  |  | ***ASIP***  **Chr. A3** | | ***MC1R***  **Chr. E2** |
|  |  |  |  |  | 24671799 | 24671780 | 63721216 |
| ***Leopardus colocolo* (pampas cat)** | | | | | | | |
| Lco-302 | Emas National Park, Goiás – Brazil | | Leandro Silveira, Anah Jacomo | Wild-type | C/T ^c,d^ | G/G | T/T |
| Lco-303 | Emas National Park, Goiás – Brazil | | Leandro Silveira, Anah Jacomo | Wild-type | C/T ^c,d^ | G/G | T/T |
| Lco-305 | Emas National Park, Goiás – Brazil | | Leandro Silveira, Anah Jacomo | Wild-type | C/T ^c,d^ | G/G | T/T |
| Lco-307 ^a^ | Emas National Park, Goiás – Brazil | | Leandro Silveira, Anah Jacomo | Wild-type | C/T ^c,d^ | G/G | T/T |
| Lco-308 ^a^ | Emas National Park, Goiás – Brazil | | Leandro Silveira, Anah Jacomo | Wild-type | C/C ^c,d^ | G/G | T/T |
| Lco-312 ^a^ | Emas National Park, Goiás – Brazil | | Leandro Silveira, Anah Jacomo | Wild-type | C/C ^c,d^ | G/G | T/T |
| Lco-313 | Emas National Park, Goiás – Brazil | | Leandro Silveira, Anah Jacomo | Wild-type | C/T ^c,d^ | G/G | T/T |
| Lco-314 ^a^ | Emas National Park, Goiás – Brazil | | Leandro Silveira, Anah Jacomo | Wild-type | C/T ^c,d^ | G/G | T/T |
| Lco-304 | Emas National Park, Goiás – Brazil | | Leandro Silveira, Anah Jacomo | Melanistic | T/T ^c,d^ | G/G | T/T |
| Lco-306 ^a^ | Emas National Park, Goiás – Brazil | | Leandro Silveira, Anah Jacomo | Melanistic | T/T ^c,d^ | G/G | T/T |
| Lco-310 | Emas National Park, Goiás – Brazil | | Leandro Silveira, Anah Jacomo | Melanistic | T/T ^c,d^ | G/G | T/T |
| Lco-311 | Emas National Park, Goiás – Brazil | | Leandro Silveira, Anah Jacomo | Melanistic | T/T ^c,d^ | G/G | T/T |
| Lco-315 | Emas National Park, Goiás – Brazil | | Leandro Silveira, Anah Jacomo | Melanistic | T/T ^c,d^ | G/G | T/T |
| Lco-316 ^a^ | Emas National Park, Goiás – Brazil | | Leandro Silveira, Anah Jacomo | Melanistic | T/T ^c,d^ | G/G | T/T |
| Lco-317 | Emas National Park, Goiás – Brazil | | Leandro Silveira, Anah Jacomo | Melanistic | T/T ^c,d^ | G/G | T/T |
| Lco-318 ^a^ | Emas National Park, Goiás – Brazil | | Leandro Silveira, Anah Jacomo | Melanistic | T/T ^c,d^ | G/G | T/T |
| Lco-320 | Emas National Park, Goiás – Brazil | | Leandro Silveira, Anah Jacomo | Melanistic | T/T ^c,d^ | G/G | T/T |
| Lco-030 ^a^ | Unkown | | Cincinnati Zoo & Botanical Garden | Melanistic | T/T ^c,d^ | G/G | T/T |
| ***Leopardus geoffroyi* (Geoffroy’s cat)** | | | | | | | |
| Lge-07 | Cachoeira do Sul, RS – Brazil | | Cachoeira do Sul Zoo | Wild-type | C/C | G/G | T/T^,d^ |
| Lge-31 | Quaraí, RS – Brazil | | Sapucaia do Sul Zoo | Wild-type | C/C | G/G | T/T ^c,d^ |
| Lge-36 ^a^ | Taim, RS – Brazil | | Tatiane Trigo | Wild-type | C/C | G/G | T/T ^c,d^ |
| Lge-37 ^b^ | São Lourenço do Sul, RS – Brazil | | Sapucaia do Sul Zoo | Wild-type | - | - | T/T ^c^ |
| Lge-46 ^a^ | Canela, RS – Brazil | | Sapucaia do Sul Zoo | Wild-type | C/C | G/G | T/T ^c,d^ |
| Lge-47 | São Leopoldo, RS - Brazil | | Sapucaia do Sul Zoo | Wild-type | - | - | T/T ^c^ |
| Lge-49 ^a^ | unknown, RS - Brazil | | Sapucaia do Sul Zoo | Wild-type | C/C | G/G | T/T ^c,d^ |
| Lge-72 | Encruzilhada do Sul, RS – Brazil | | Quinta da Estância Grande | Wild-type | C/C | G/G | T/T ^d^ |
| Lge-73 | Cachoeira do Sul, RS – Brazil | | Cachoeira Zoo | Wild-type | C/C | G/G | T/T ^c,d^ |
| Lge-75 | Arroio Grande, RS – Brazil | | Fabio Mazim e José Bonifácio Garcia Soares | Wild-type | C/C | G/G | T/T ^c,d^ |
| Lge-78 | Rio Grande, RS – Brazil | | Fabio Mazim | Wild-type | C/C | G/G | T/T^,d^ |
| Lge-92 ^a^ | Alegrete, RS – Brazil | | Fundação Zoobotânica do Rio Grande do Sul | Wild-type | C/C | G/G | T/T^,d^ |
| Lge-93 | Arroio Grande, RS – Brazil | | Fundação Zoobotânica do Rio Grande do Sul | Wild-type | C/C | G/G | T/T ^c,d^ |
| Lge-94 | Vale do Quilombo, RS – Brazil | | Fundação Zoobotânica do Rio Grande do Sul | Wild-type | C/C | G/G | T/T ^c,d^ |
| Lge-95 | Cristal, RS – Brazil | | Fundação Zoobotânica do Rio Grande do Sul | Wild-type | C/C | G/G | T/T ^d^ |
| Lge-96 | Pelotas, RS – Brazil | | Fundação Zoobotânica do Rio Grande do Sul | Wild-type | C/C | G/G | T/T ^d^ |
| Lge-01 | Santa Cruz do Sul, RS – Brazil | | Sapucaia do Sul Zoo | Melanistic | C/C | G/G | C/T ^c,d^ |
| Lge-04 | Cachoeira do Sul, RS – Brazil | | Cachoeira do Sul Zoo | Melanistic | C/C | G/G | C/T ^c,d^ |
| Lge-29 ^a^ | Quaraí, RS – Brazil | | Dênis Sana, Tatiane Trigo, Cibele Indrusiak | Melanistic | C/C | G/G | C/T ^c,d^ |
| Lge-71 | Pelotas, RS – Brazil | | Thales R. O. de Freitas e José Stoltz | Melanistic | C/C | G/G | C/T ^c,d^ |
| Lge-74 ^a^ | Pinheiro Machado, RS – Brazil | | Fabio Mazim | Melanistic | C/C | G/G | C/T ^c,d^ |
| Lge-77 ^a^ | Dom Pedrito, RS – Brazil | | Fabio Mazim | Melanistic | C/C | G/G | C/T ^c,d^ |
| Lge-91 ^a^ | Itaqui, RS – Brazil | | Fundação Zoobotânica do Rio Grande do Sul | Melanistic | C/C | G/G | C/T ^d^ |
| ***Leopardus guigna* (kodkod)** | | | | | | | |
| Lgu-18 ^b^ | Chiloé Island, Chile | Constanza Napolitano | | Wild-type | C/C | G/G ^c^ | - |
| Lgu-19 ^a^ | Chiloé Island, Chile | Constanza Napolitano | | Wild-type | C/C | G/A ^c,d^ | T/T |
| Lgu-21 | Chiloé Island, Chile | Constanza Napolitano | | Wild-type | C/C | G/G ^c,d^ | T/T |
| Lgu-22 | Chiloé Island, Chile | Constanza Napolitano | | Wild-type | C/C | G/G ^c,d^ | T/T |
| Lgu-76 | Chiloé Island, Chile | Constanza Napolitano | | Wild-type | C/C | G/G ^c,d^ | T/T |
| Lgu-77 ^a^ | Chiloé Island, Chile | Constanza Napolitano | | Wild-type | C/C | G/A ^c,d^ | T/T |
| Lgu-78 ^a^ | Chiloé Island, Chile | Constanza Napolitano | | Wild-type | C/C | G/G ^c,d^ | T/T |
| Lgu-80 | Chiloé Island, Chile | Constanza Napolitano | | Wild-type | C/C | G/A ^c,d^ | T/T |
| Lgu-81 | Chiloé Island, Chile | Constanza Napolitano | | Wild-type | C/C | G/A ^c,d^ | TT |
| Lgu-82 | Chiloé Island, Chile | Constanza Napolitano | | Wild-type | C/C | G/A ^c,d^ | T/T |
| Lgu-85 | Chiloé Island, Chile | Constanza Napolitano | | Wild-type | C/C | G/A ^c,d^ | T/T |
| Lgu-24 ^a^ | Chiloé Island, Chile | Constanza Napolitano | | Melanistic | C/C | A/A ^c,d^ | T/T |
| Lgu-73 ^a,b^ | Chiloé Island, Chile | Constanza Napolitano | | Melanistic | C/C | A/A ^c^ | T/T |
| Lgu-74 | Chiloé Island, Chile | Constanza Napolitano | | Melanistic | C/C | A/A ^c,d^ | T/T |
| Lgu-75 ^a^ | Chiloé Island, Chile | Constanza Napolitano | | Melanistic | C/C | A/A ^c,d^ | T/T |
| Lgu-79 ^b^ | Chiloé Island, Chile | Constanza Napolitano | | Melanistic | C/C | A/A ^c^ | - |

^a^ Samples selected for the initial PCR characterization of *Asip* and *Mc1r* (four individuals of each phenotype from each individual species).

^b^ Samples that were excluded from the hybrid capture assay due to insufficient DNA.

^c^ Determined by Sanger sequencing.

^d^ Determined by next-generation sequencing.

**Table S2. Primers used for PCR amplification and sequencing of *MC1R* in felids*.* F1/R1 refer to the first fragment of the coding exon, while F2/R2 refer to the second fragment of the coding exon.**

| **Primer ID** | **Primer sequence (5’ – 3’)** |
| --- | --- |
| *MC1R* – F1 | CCTGCTGGAAGCACCACT |
| *MC1R* – R1 | GACGCTAGCCACCCAGATAG |
| *MC1R* – F2 | GTGGACCGCTACATTTCCAT |
| *MC1R* – R2 | GCCATAGGATATCCCCACCT |

**Table S3. GenBank accession numbers of mammalian sequences for comparative genomic analyses of *ASIP* and *MC1R* genes.**

| **Species** | | **GenBank accession number** | |
| --- | --- | --- | --- |
|  |  | ***Asip*** | ***Mc1r*** |
| Domestic cat (*Felis catus*) | NP_001009190.1 | | AY237395 |
| Dog (*Canis familiaris*) | NP_001007264.1 | | AF064455 |
| Human (*Homo sapiens*) | NM_001672 | | AF326275 |
| Cow (*Bos taurus*) | X99692.1 | | GU982927 |
| Mouse (*Mus musculus*) | NM_015770.3 | | AB306322 |
| Red fox (*Vulpes vulpes*) | -- | | X90844 |
| Horse (*Equus caballus*) | AF288358.1 | | -- |
| Pig (*Sus scrofa*) | AJ427478.2 | | -- |
| Rat (*Rattus rattus*) | NM_052979.1 | | -- |

**Table S4. *ASIP* and *MC1R* coding sequence variants identified in discovery panel (4 individuals of each phenotype). Melanistic individuals and their respective genotypes indicated in bold.**

| *ASIP* (coordinates on chr. A3) | | | | | | | *MC1R* variable loci (coordinates on chr. E2) | | | | | | | | | | | |
| --- | --- | --- | --- | --- | --- | --- | --- | --- | --- | --- | --- | --- | --- | --- | --- | --- | --- | --- |
|  | 2 | 2 | 2 | 2 | **2** | **2** | 6 | 6 | 6 | 6 | **6** | 6 | 6 | 6 | 6 | 6 | 6 | 6 |
|  | 4 | 4 | 4 | 4 | **4** | **4** | 3 | 3 | 3 | 3 | **3** | 3 | 3 | 3 | 3 | 3 | 3 | 3 |
|  | 6 | 6 | 6 | 6 | **6** | **6** | 7 | 7 | 7 | 7 | **7** | 7 | 7 | 7 | 7 | 7 | 7 | 7 |
|  | 7 | 7 | 7 | 7 | **7** | **7** | 2 | 2 | 2 | 2 | **2** | 2 | 2 | 2 | 2 | 2 | 2 | 2 |
|  | 6 | 6 | 1 | 1 | **1** | **1** | 0 | 0 | 0 | 0 | **1** | 1 | 1 | 1 | 1 | 1 | 1 | 1 |
|  | 0 | 0 | 8 | 8 | **7** | **7** | 8 | 9 | 9 | 9 | **2** | 2 | 3 | 3 | 4 | 4 | 7 | 7 |
|  | 9 | 8 | 6 | 0 | **9** | **8** | 6 | 0 | 2 | 4 | **1** | 1 | 7 | 7 | 2 | 3 | 0 | 1 |
|  | 6 | 6 | 4 | 3 | **9** | **0** | 3 | 6 | 4 | 0 | **6** | 8 | 3 | 4 | 3 | 4 | 2 | 0 |
| *F. catus* | C | G | C | A | C | G | A | C | C | C | T | T | C | G | G | A | A | C |
| Lco-307 | T | A | T | **.** | C | **.** | N | G | **.** | C/T | **.** | **.** | **.** | **.** | A | C | **.** | **.** |
| Lco-308 | T | A | T | **.** | C/T | **.** | **.** | G | **.** | **.** | **.** | **.** | **.** | **.** | A | C | **.** | **.** |
| Lco-312 | T | A | T | **.** | C | **.** | N | **.** | **.** | **.** | **.** | **.** | **.** | **.** | A | C | **.** | **.** |
| Lco-314 | T | A | T | **.** | C/T | **.** | C | **.** | T | **.** | **.** | **.** | **.** | **.** | A | C | **.** | **.** |
| Lco-030 | T | A | T | **.** | **T** | **.** | C | **.** | **.** | **.** | **.** | **.** | **.** | **.** | A | C | **.** | **.** |
| Lco-306 | T | A | T | **.** | **T** | **.** | C/A | **.** | **.** | **.** | **.** | **.** | **.** | **.** | A | C | **.** | **.** |
| Lco-316 | T | A | T | **.** | **T** | **.** | C/A | **.** | **.** | **.** | **.** | **.** | **.** | **.** | A | C | **.** | **.** |
| Lco-318 | T | A | T | **.** | **T** | **.** | C/A | G | **.** | **.** | **.** | **.** | **.** | **.** | A | C | **.** | **.** |
|  |  |  |  |  |  |  |  |  |  |  |  |  |  |  |  |  |  |  |
| Lgu-19 | **.** | **.** | **.** | **.** | **.** | G/A | **.** | **.** | **.** | **.** | **.** | **.** | **.** | **.** | **.** | **.** | G | **.** |
| Lgu-77 | **.** | **.** | **.** | **.** | **.** | G/A | **.** | **.** | **.** | **.** | **.** | **.** | **.** | **.** | **.** | **.** | G | **.** |
| Lgu-78 | **.** | **.** | **.** | **.** | **.** | G | **.** | **.** | **.** | **.** | **.** | **.** | **.** | **.** | **.** | **.** | G | **.** |
| Lgu-81 | **.** | **.** | **.** | **.** | **.** | G/A | **.** | **.** | **.** | **.** | **.** | **.** | **.** | **.** | **.** | **.** | G | **.** |
| Lgu-24 | **.** | **.** | **.** | **.** | **.** | **A** | **.** | **.** | **.** | **.** | **.** | **.** | **.** | **.** | **.** | **.** | G | **.** |
| Lgu-73 | **.** | **.** | **.** | **.** | **.** | **A** | **.** | **.** | **.** | N | **.** | **.** | **.** | **.** | **.** | **.** | G | **.** |
| Lgu-75 | **.** | **.** | **.** | **.** | **.** | **A** | **.** | **.** | **.** | **.** | **.** | **.** | **.** | **.** | **.** | **.** | G | **.** |
| Lgu-79 | **.** | **.** | **.** | **.** | **.** | **A** | **.** | **.** | **.** | **.** | **.** | **.** | **.** | **.** | **.** | **.** | G | **.** |
|  |  |  |  |  |  |  |  |  |  |  |  |  |  |  |  |  |  |  |
| Lge-36 | **.** | **.** | **.** | A/G | **.** | **.** | **.** | **.** | **.** | **.** | **.** | C/T | **.** | **.** | **.** | **.** | G | C/T |
| Lge-46 | **.** | **.** | **.** | **.** | **.** | **.** | **.** | **.** | **.** | **.** | **.** | **.** | **.** | **.** | **.** | **.** | G | **.** |
| Lge-49 | **.** | **.** | **.** | **.** | **.** | **.** | **.** | **.** | **.** | **.** | **.** | **.** | **.** | **.** | **.** | **.** | G | **.** |
| Lge-92 | **.** | **.** | **.** | **.** | **.** | **.** | **.** | **.** | **.** | **.** | **.** | **.** | **.** | **.** | **.** | **.** | G | C/T |
| Lge-29 | **.** | **.** | **.** | **.** | **.** | **.** | **.** | **.** | **.** | **.** | **C/T** | **.** | C/T | A/G | A/G | **.** | G | **.** |
| Lge-74 | **.** | **.** | **.** | **.** | **.** | **.** | **.** | **.** | **.** | **.** | **C/T** | **.** | N | N | N | **.** | G | **.** |
| Lge-77 | **.** | **.** | **.** | **.** | **.** | **.** | **.** | **.** | **.** | **.** | **C/T** | **.** | C/T | A/G | A/G | **.** | G | **.** |
| Lge-91 | **.** | **.** | **.** | **.** | **.** | **.** | **.** | **.** | **.** | **.** | **N** | **.** | C/T | A/G | A/G | **.** | G | **.** |

**Table S5. *ASIP* coding sequence variants in entire set of pampas cat and kodkod samples. Melanistic individuals and their respective genotypes indicated in bold.**

| **Animal** | **Coordinate (chr. A3)** | | |
| --- | --- | --- | --- |
|  | 2 | **2** | **2** |
|  | 4 | **4** | **4** |
|  | 6 | **6** | **6** |
|  | 7 | **7** | **7** |
|  | 1 | **1** | **1** |
|  | 8 | **7** | **7** |
|  | 6 | **9** | **8** |
|  | 4 | **9** | **0** |
| ***F. catus*** | C | C | G |
| Lco-302 | T | C/T | **.** |
| Lco-303 | T | C/T | **.** |
| Lco-305 | T | C/T | **.** |
| Lco-307 | T | C/T | **.** |
| Lco-308 | T | C | **.** |
| Lco-312 | T | C | **.** |
| Lco-313 | T | C/T | **.** |
| Lco-314 | T | C/T | **.** |
| **Lco-030** | T | **T** | **.** |
| **Lco-304** | T | **T** | **.** |
| **Lco-306** | T | **T** | **.** |
| **Lco-310** | T | **T** | **.** |
| **Lco-311** | T | **T** | **.** |
| **Lco-315** | T | **T** | **.** |
| **Lco-316** | T | **T** | **.** |
| **Lco-317** | T | **T** | **.** |
| **Lco-318** | T | **T** | **.** |
| **Lco-320** | T | **T** | **.** |
| Lgu-18 | **.** | **.** | **.** |
| Lgu-19 | **.** | **.** | A/G |
| Lgu-21 | **.** | **.** | **.** |
| Lgu-22 | **.** | **.** | **.** |
| Lgu-76 | **.** | **.** | **.** |
| Lgu-77 | **.** | **.** | A/G |
| Lgu-78 | **.** | **.** | **.** |
| Lgu-80 | **.** | **.** | A/G |
| Lgu-81 | **.** | **.** | A/G |
| Lgu-82 | **.** | **.** | A/G |
| Lgu-85 | **.** | **.** | A/G |
| **Lgu-24** | **.** | **.** | **A** |
| **Lgu-73** | **.** | **.** | **A** |
| **Lgu-74** | **.** | **.** | **A** |
| **Lgu-75** | **.** | **.** | **A** |
| **Lgu-79** | **.** | **.** | **A** |

**Table S6. *MC1R* coding sequence variants in entire set of Geoffroy’s cat samples. Melanistic individuals and their respective genotypes indicated in bold.**

| **Animal** | **Coordinate (chr. E2)** | | | | | |
| --- | --- | --- | --- | --- | --- | --- |
|  | **6** | 6 | 6 | 6 | 6 | 3 |
|  | **3** | 3 | 3 | 3 | 3 | 3 |
|  | **7** | 7 | 7 | 7 | 7 | 7 |
|  | **2** | 2 | 2 | 2 | 2 | 2 |
|  | **1** | 1 | 1 | 1 | 1 | 1 |
|  | **2** | 2 | 3 | 3 | 4 | 7 |
|  | **1** | 1 | 7 | 7 | 2 | 1 |
|  | **6** | 8 | 3 | 4 | 3 | 0 |
| ***F. catus*** | T | T | C | G | G | C |
| Lge-07 | N | N | N | N | N | N |
| Lge-31 | **.** | **.** | N | N | N | **.** |
| Lge-36 | **.** | C/T | **.** | **.** | **.** | C/T |
| Lge-37 | **.** | **.** | **.** | **.** | **.** | **.** |
| Lge-46 | **.** | **.** | **.** | **.** | **.** | **.** |
| Lge-47 | **.** | **.** | **.** | **.** | **.** | **.** |
| Lge-49 | **.** | **.** | **.** | **.** | **.** | **.** |
| Lge-72 | N | N | N | N | N | N |
| Lge-73 | **.** | **.** | **.** | **.** | **.** | **.** |
| Lge-75 | **.** | **.** | **.** | **.** | **.** | **.** |
| Lge-78 | N | N | N | N | N | N |
| Lge-92 | N | N | **.** | **.** | **.** | C/T |
| Lge-93 | **.** | **.** | **.** | **.** | **.** | **.** |
| Lge-94 | **.** | **.** | **.** | **.** | **.** | **.** |
| Lge-95 | N | N | N | N | N | **.** |
| **Lge-01** | **C/T** | **.** | C/T | A/G | A/G | **.** |
| **Lge-04** | **C/T** | **.** | N | N | N | **.** |
| **Lge-29** | **C/T** | **.** | C/T | A/G | A/G | **.** |
| **Lge-71** | **C/T** | **.** | N | N | N | **.** |
| **Lge-74** | **C/T** | **.** | N | N | N | **.** |
| **Lge-77** | **C/T** | **.** | C/T | A/G | A/G | **.** |
| **Lge-91** | **N** | N | C/T | A/G | A/G | **.** |

**Table S7. Chromosome coordinates of *F. catus* fosmid clones selected for probe synthesis and targeted resequencing of *ASIP* and *MC1R* regions.**

| Segment | Fosmid ID | Chromosomal coordinates |
| --- | --- | --- |
| AsipA | 165060_F.CATUS_FOSMID-042_M13 | chrA3(-): 25056649-25024544 |
|  | 161571_F.CATUS_FOSMID-095_K15 | chrA3(-): 25045115-25007212 |
| AsipB | 165209_F.CATUS_FOSMID-082_C20 | chrA3(-): 24823580-24791354 |
| AsipC | 162081_ F.CATUS_FOSMID-14_C16 | chrA3(-): 24758740-24718737 |
| AsipD | 162155_F.CATUS_FOSMID-036_A21 | chrA3(-): 24682385-24647588 |
| AsipE | 162683_F.CATUS_FOSMID-072_H5 | chrA3(-): 24581617-24539008 |
| AsipF | 161863_F.CATUS_FOSMID-013_F1 | chrA3(-): 24425210-24387945 |
| AsipG | 161280_F.CATUS_FOS-003_J17 | chrA3(-): 24372035-24335850 |
| AsipH | 161571_F.CATUS_FOSMID-075_I22 | chrA3(-): 24253356-24215096 |
| Mc1rA | 162683_F.CATUS_FOSMID-039_H22 | chrE2(+): 63479041-63518921 |
|  | 162298_F.CATUS_FOSMID-035_E17 | chrE2(+): 63500324-63531473 |
| Mc1rB | 161957_F.CATUS_FOSMID-032_P20 | chrE2(+): 63651257-63686910 |
| Mc1rC | 165209_F.CATUS_FOSMID-058_N1 | chrE2(+): 63688765-63722767 |
|  | 161795_F.CATUS_FOS-044_G13 | chrE2(+): 63703586-63740307 |
|  | 162155_F.CATUS_FOSMID-093_J7 | chrE2(+): 63723969-63763339 |
| Mc1rD | 165232_F.CATUS_FOSMID-028_C14 | chrE2(+): 63761683-63796695 |
| Mc1rE | 162683_F.CATUS_FOSMID-081_M17 | chrE2(+): 63811726-63846524 |
|  | 162683_F.CATUS_FOSMID-044_K8 | chrE2(+): 63849049-63887899 |

**Table S8. Sequence statistics for *ASIP* and *MC1R* genomic segments in the pampas cat (Lco), Geoffroy’s cat (Lge) and kodkod (Lgu), and the corresponding number of variants identified in each species.**

| Segment | Length (bp) | Unknown  bp (%) | Masked repeats  bp (%) | Number of altered loci in consensus sequences - bp (%) | | | Number of SNPs | | |
| --- | --- | --- | --- | --- | --- | --- | --- | --- | --- |
|  |  |  |  | **Lco** | **Lge** | **Lgu** | **Lco** | **Lge** | **Lgu** |
| AsipA | 49437 | 208 (0.42) | 20817 (42.11) | 465 (1.62) | 424 (1.48) | 453 (1.58) | 49 | 49 | 15 |
| AsipB | 20667 | 243 (1.18) | 9326 (45.13) | 171 (1.51) | 163 (1.44) | 162 (1.43) | 8 | 12 | 5 |
| AsipC | 40003 | 569 (1.42) | 22001 (55) | 281 (1.56) | 257 (1.43) | 282 (1.57) | 13 | 40 | 6 |
| AsipD | 34797 | 40 (0.11) | 12737 (36.6) | 431 (1.95) | 417 (1.89) | 435 (1.97) | 16 | 35 | 10 |
| AsipE | 42609 | 313 (0.73) | 28540 (66.98) | 144 (1.02) | 117 (0.83) | 128 (0.91) | 19 | 22 | 5 |
| AsipF | 37265 | 20 (0.05) | 18094 (48.55) | 263 (1.37) | 240 (1.25) | 251 (1.31) | 28 | 37 | 7 |
| AsipG | 36185 | 338 (0.93) | 20169 (55.74) | 181 (1.13) | 173 (1.08) | 184 (1.15) | 17 | 36 | 11 |
| AsipH | 38260 | 810 (2.12) | 17332 (45.3) | 237 (1.13) | 214 (1.02) | 224 (1.07) | 6 | 13 | 0^*^ |
| *ASIP* | **299223** | **2541 (0.85)** | **149016 (49.8)** | **2173 (1.45)** | **2005 (1.33)** | **2119 (1.41)** | **156** | **244** | **59** |
| Mc1rA | 31838 | 20 (0.06) | 3399 (10.68) | 810 (2.85) | 767 (2.7) | 794 (2.79) | 32 | 32 | 20 |
| Mc1rB | 33790 | 1247 (3.69) | 8505 (25.17) | 626 (2.48) | 589 (2.33) | 618 (2.44) | 33 | 33 | 10 |
| Mc1rC | 74574 | 274 (0.37) | 21137 (28.34) | 1000 (1.87) | 956 (1.79) | 1033 (1.93) | 51 | 54 | 13 |
| Mc1rD | 35012 | 370 (1.06) | 10858 (31.01) | 607 (2.51) | 600 (2.48) | 650 (2.69) | 35 | 35 | 24 |
| Mc1rE | 76173 | 107 (0.14) | 27875 (36.59) | 1042 (2.16) | 993 (2.06) | 1059 (2.19) | 76 | 76 | 23 |
| *MC1R* | **251387** | **2018 (0.8)** | **71774 (28.55)** | **4085 (2.27)** | **3905 (2.17)** | **4154 (2.31)** | **227** | **230** | **90** |

^*^No variants were identified on AsipH in kodkod samples.

**Table S9. Alignment statistics.**

| Species | *F. catus* genome | Unmasked *ASIP* and *MC1R* fragments | Masked *ASIP* and *MC1R* fragments |
| --- | --- | --- | --- |
| Pampas cat | 69.45% | 40.77% | 4.83% |
| Geoffroy’s cat | 63.84% | 35.53% | 3.75% |
| Kodkod | 64.75% | 38.16% | 4.57% |

**Table S10. Coverage statistics for each segment.**

| Segment | Pampas cat | | | | | Geoffroy’s cat | | | | Kodkod | | | | |
| --- | --- | --- | --- | --- | --- | --- | --- | --- | --- | --- | --- | --- | --- | --- |
|  | **Unmasked** | | **Masked** | | | **Unmasked** | | **Masked** | | **Unmasked** | | **Masked** | | |
|  | **Mean** | **>10X** | **Mean** | | **>10X** | **Mean** | **>10X** | **Mean** | **>10X** | **Mean** | **>10X** | **Mean** | | **>10X** |
| AsipA | 247.47 | 99.69 | 22.54 | 94.96 | | 118.98 | 99.30 | 15.13 | 93.15 | 284.64 | 99.72 | | 39.83 | 96.12 |
| AsipB | 117.71 | 89.47 | 20.49 | 87.92 | | 60.04 | 86.61 | 13.22 | 80.40 | 139.02 | 89.43 | | 35.57 | 89.01 |
| AsipC | 92.23 | 98.81 | 22.14 | 94.55 | | 46.87 | 94.75 | 14.39 | 88.78 | 107.17 | 99.15 | | 38.75 | 96.02 |
| AsipD | 199.39 | 99.88 | 23.13 | 97.40 | | 98.13 | 99.62 | 15.41 | 94.52 | 225.49 | 99.92 | | 41.06 | 98.29 |
| AsipE | 159.62 | 99.62 | 20.96 | 89.88 | | 87.39 | 98.84 | 14.07 | 87.05 | 200.23 | 99.91 | | 36.51 | 91.40 |
| AsipF | 247.17 | 99.62 | 22.93 | 96.71 | | 124.11 | 98.62 | 15.17 | 93.36 | 293.43 | 99.80 | | 40.63 | 97.41 |
| AsipG | 184.36 | 96.94 | 22.00 | 92.70 | | 98.77 | 97.09 | 14.49 | 88.82 | 224.97 | 98.34 | | 39.04 | 93.30 |
| AsipH | 149.47 | 99.81 | 22.32 | 94.35 | | 80.48 | 98.34 | 14.70 | 90.63 | 179.23 | 99.82 | | 39.09 | 95.33 |
| Mc1rA | 193.92 | 99.66 | 23.69 | 99.33 | | 81.37 | 97.57 | 15.72 | 97.64 | 216.00 | 99.84 | | 42.05 | 99.72 |
| Mc1rB | 142.02 | 98.12 | 22.79 | 96.17 | | 64.54 | 94.06 | 15.01 | 92.33 | 161.19 | 98.25 | | 40.04 | 97.23 |
| Mc1rC | 123.40 | 74.20 | 17.38 | 73.55 | | 54.20 | 71.97 | 11.49 | 70.91 | 139.00 | 74.37 | | 30.69 | 74.16 |
| Mc1rD | 134.07 | 97.96 | 22.59 | 95.29 | | 60.35 | 95.82 | 15.10 | 93.47 | 148.63 | 99.19 | | 40.27 | 97.47 |
| Mc1rE | 208.28 | 96.46 | 22.07 | 93.04 | | 110.68 | 98.03 | 15.32 | 94.39 | 247.50 | 99.09 | | 40.46 | 97.36 |

**Table S11. Nucleotide diversity within each species according to fosmid and phenotype (SEM).**

|  | **Pampas cat** | | | |  | | **Geoffroy’s cat** | | | |  | | **Kodkod** | | | |  |
| --- | --- | --- | --- | --- | --- | --- | --- | --- | --- | --- | --- | --- | --- | --- | --- | --- | --- |
| **Fosmid** | | **NM** | **M** | **All** | |  | | **NM** | **M** | **All** | |  | | **NM** | **M** | **All** | |
| **AsipA** | | 0.51 (0.07) | 0.55 (0.06) | 0.56 (0.05) | |  | | 0.74 (0.07) | 0.8 (0.11) | 0.76 (0.06) | |  | | 0.18 (0.02) | 0.16 (0.04) | 0.18 (0.02) | |
| **AsipB** | | 0.23 (0.03) | 0.2 (0.03) | 0.22 (0.02) | |  | | 0.34 (0.03) | 0.34 (0.05) | 0.34 (0.03) | |  | | 0.2 (0.03) | 0.22 (0.06) | 0.19 (0.02) | |
| **AsipC** | | 0.24 (0.03) | 0.2 (0.02) | 0.21 (0.02) | |  | | 0.85 (0.08) | 0.99 (0.14) | 0.89 (0.07) | |  | | 0.16 (0.02) | 0.06 (0.02) | 0.16 (0.02) | |
| **AsipD** | | 0.31 (0.04) | 0.02 (0) | 0.18 (0.02) | |  | | 0.71 (0.07) | 0.66 (0.09) | 0.69 (0.05) | |  | | 0.16 (0.02) | 0.02 (0.01) | 0.18 (0.02) | |
| **AsipE** | | 0.59 (0.08) | 0.16 (0.02) | 0.4 (0.03) | |  | | 0.58 (0.06) | 0.6 (0.08) | 0.59 (0.05) | |  | | 0.06 (0.01) | 0.18 (0.05) | 0.09 (0.01) | |
| **AsipF** | | 0.56 (0.07) | 0.42 (0.05) | 0.51 (0.04) | |  | | 0.67 (0.06) | 0.71 (0.1) | 0.67 (0.05) | |  | | 0.08 (0.01) | 0.18 (0.05) | 0.12 (0.01) | |
| **AsipG** | | 0.39 (0.05) | 0.33 (0.04) | 0.36 (0.03) | |  | | 0.72 (0.07) | 0.81 (0.11) | 0.77 (0.06) | |  | | 0.25 (0.03) | 0.26 (0.07) | 0.25 (0.03) | |
| **AsipH** | | 0.12 (0.02) | 0.07 (0.01) | 0.1 (0.01) | |  | | 0.23 (0.02) | 0.23 (0.03) | 0.23 (0.02) | |  | | 0 | 0 | 0 | |
| ***ASIP*** | | 0.42 (0.07) | 0.27 (0.03) | 0.33 (0.03) | |  | | 0.64 (0.05) | 0.54 (0.11) | 0.63 (0.05) | |  | | 0.14 (0.02) | 0.14 (0.02) | 0.17 (0.02) | |
|  | |  |  |  | |  | |  |  |  | |  | |  |  |  | |
| **Mc1rA** | | 0.52 (0.07) | 0.47 (0.06) | 0.49 (0.04) | |  | | 0.47 (0.05) | 0.43 (0.06) | 0.45 (0.04) | |  | | 0.25 (0.03) | 0.29 (0.07) | 0.28 (0.03) | |
| **Mc1rB** | | 0.63 (0.08) | 0.63 (0.07) | 0.63 (0.05) | |  | | 0.51 (0.05) | 0.29 (0.04) | 0.44 (0.03) | |  | | 0.15 (0.02) | 0.17 (0.04) | 0.15 (0.02) | |
| **Mc1rC** | | 0.37 (0.05) | 0.43 (0.05) | 0.41 (0.03) | |  | | 0.4 (0.04) | 0.34 (0.05) | 0.39 (0.03) | |  | | 0.08 (0.01) | 0.08 (0.02) | 0.08 (0.01) | |
| **Mc1rD** | | 0.66 (0.09) | 0.65 (0.07) | 0.66 (0.06) | |  | | 0.66 (0.06) | 0.59 (0.08) | 0.63 (0.05) | |  | | 0.33 (0.04) | 0.34 (0.08) | 0.32 (0.03) | |
| **Mc1rE** | | 0.59 (0.08) | 0.67 (0.08) | 0.65 (0.05) | |  | | 0.68 (0.06) | 0.71 (0.1) | 0.69 (0.05) | |  | | 0.2 (0.02) | 0.24 (0.06) | 0.21 (0.02) | |
| ***MC1R*** | | 0.53 (0.09) | 0.56 (0.05) | 0.55 (0.04) | |  | | 0.52 (0.04) | 0.51 (0.11) | 0.52 (0.04) | |  | | 0.19 (0.03) | 0.19 (0.03) | 0.19 (0.02) | |
|  | |  |  |  | |  | |  |  |  | |  | |  |  |  | |
| **Both Loci** | | 0.48 (0.08) | 0.42 (0.04) | **0.45 (0.04)** | |  | | 0.17 (0.02) | 0.16 (0.02) | **0.18 (0.02)** | |  | | 0.58 (0.05) | 0.52 (0.11) | **0.57 (0.04)** | |
